# Characterizing Fibrosis and Inflammation in a Partial Bile Duct Ligation Mouse Model by Multiparametric Magnetic Resonance Imaging

**DOI:** 10.1002/jmri.27925

**Published:** 2021-09-21

**Authors:** Jia‐Yi Liu, Ye‐Yu Cai, Zhu‐Yuan Ding, Zi‐Yi Zhou, Min Lv, Huan Liu, Li‐Yun Zheng, Lan Li, Yong‐Heng Luo, En‐Hua Xiao

**Affiliations:** ^1^ Department of Radiology The Second Xiangya Hospital of Central South University Changsha China; ^2^ MR Collaboration, Central Research Institute United Imaging Healthcare Shanghai China; ^3^ Department of Pathology The Second Xiangya Hospital of Central South University Changsha China; ^4^ Medical Imaging Research Center Central South University Changsha 410008 China

**Keywords:** bile duct, liver, fibrosis, magnetic resonance imaging, mice

## Abstract

**Background:**

Partial bile duct ligation (PBDL) model is a reliable cholestatic fibrosis experimental model that showed complex histopathological changes. Magnetic resonance imaging (MRI) features of PBDL have not been well characterized.

**Purpose:**

To investigate the potential of MRI parameters in assessing fibrosis in PBDL and explore the relationships between MRI and pathological features.

**Animal Model:**

Established PBDL models.

**Population:**

Fifty‐four mice were randomly divided into four timepoints PBDL groups and one sham group.

**Field Strength/Sequence:**

3.0 T; MRI sequences included T1‐weighted fast spin‐echo (FSE), T2‐weighted single shot FSE, variable flip angle T1 mapping, multi‐echo SE T2 mapping, multi‐echo gradient‐echo T2* mapping, and multi‐*b*‐value diffusion‐weighted imaging.

**Assessment:**

MRI examination was performed at the corresponding timepoints after surgery. Native T1, ΔT1 (T1native‐T1post), T2, T2*, apparent diffusion coefficient (ADC) values, histogram parameters (skewness and kurtosis), intravoxel incoherent motion parameters (*f*, *D*, and *D* ^*^) within the entire ligated (PBDL), non‐ligated liver (PBDL), and whole liver (sham) were obtained. Fibrosis and inflammation were assessed in Masson and H&E staining slices using the Metavir and activity scoring system.

**Statistical Tests:**

One‐way ANOVA, Spearman's rank correlation, and receiver operating characteristic curves were performed. *P* < 0.05 was considered statistically significant.

**Results:**

Fibrosis and inflammation were finally staged as F3 and A3 in ligated livers but were not observed in non‐ligated or sham livers. Ligated livers displayed significantly elevated native T1, ΔT1, T2, and reduced ADC and T2^*^ than other livers. Spearman's correlation showed better correlation with inflammation (*r* = 0.809) than fibrosis (*r* = 0.635) in T2 and both ΔT1 and ADC showed stronger correlation with fibrosis (*r* = 0.704 and *r* = −0.718) than inflammation (*r* = 0.564 and *r* = −0.550). Area under the curve (AUC) for ΔT1 performed the highest (0.896). When combined with all relative parameters, AUC increased to 0.956.

**Data Conclusion:**

Multiparametric MRI can evaluate and differentiate pathological changes in PBDL. ΔT1 and ADC better correlated with fibrosis while T2 stronger with inflammation.

**Level of Evidence:**

1

**Technical Efficacy:**

Stage 2

Liver fibrosis is the accumulation of extracellular matrix proteins that replace the damaged normal tissue.^1^ Cholestatic fibrosis results from obstruction to bile flow, primary or secondary biliary cholangitis, primary sclerosing cholangitis, or biliary atresia.^2^ Depending on etiologies, liver fibrosis is associated with several main histopathological processes, including injury, inflammation, and collagen accumulation.^3^

Surgical models of cholestasis, that is, common bile duct ligation (CBDL), have been widely used in the past decades.^4^, ^5^ Although CBDL causes obstructive cholestasis, inflammation, and fibrosis postoperatively, this procedure may require more animals than expected due to the higher mortality rates (over 50%).^6^ In laboratory animal studies, 3R principles,^7^ which guide the ethical handling of laboratory animals, should be followed. This may be achieved by refining the procedures in favor of animal welfare,^8^ reducing the number of animals needed,^9^ and replacing animal models.^10^ Partial bile duct ligation (PBDL) is favorable not only to the 3R principles by keeping the liver function intact to decrease morbidity and mortality,^6^ but also the internal comparisons between the ligated and non‐ligated lobes, which are subjected to the same systemic effects, such as the biological internal environment.

Noninvasive imaging approaches that provide whole‐body images are broadly used in laboratory studies to describe morphological and histopathological processes.^11^ Magnetic resonance imaging (MRI) is a non‐invasive method with excellent spatial resolution.^12^ Laboratory animals have been shown to well tolerate imaging techniques.^13^ Several recent studies only found the single association between MRI features and hepatic fibrosis.^14^, ^15^ However, the histopathological changes of liver fibrosis are complex. Studies have not been well characterized using MRI to differentiate the complex histopathological changes. Therefore, it is essential to establish a comprehensive experimental system that comprises both reproducible cholestatic models and reliable MR imaging techniques to fully exploit the potential of noninvasive cholestatic fibrosis process monitoring.

## Materials and Methods

### Animals and Study Design

All procedures and protocols were approved by the animal care committee of the Second Xiangya Hospital of Central South University (No. 2020495). A total of 54 8‐week‐old male BALB/C mice obtained from Animal Laboratory of the Second Xiangya Hospital were used for this study.

Mice were divided into the PBDL (*N* = 48) and sham (*N* = 6) groups. To characterize the imaging and histopathologic changes of the PBDL model, postoperative 2, 4, 6, and 8 weeks were designated as four different timepoint subgroups, with 12 mice in each subgroup. All mice received multiparametric MRI examination at the end of their assigned timepoint. Subsequently, mice were killed to measure the body weight and the liver weight (g) for histopathologic analysis. To characterize the development of liver injury after PBDL, we compared the mouse body weight, liver weight, and histological features of fibrosis and inflammation between ligated, non‐ligated, and sham groups over time.

### PBDL Surgery

**FIGURE 1:**
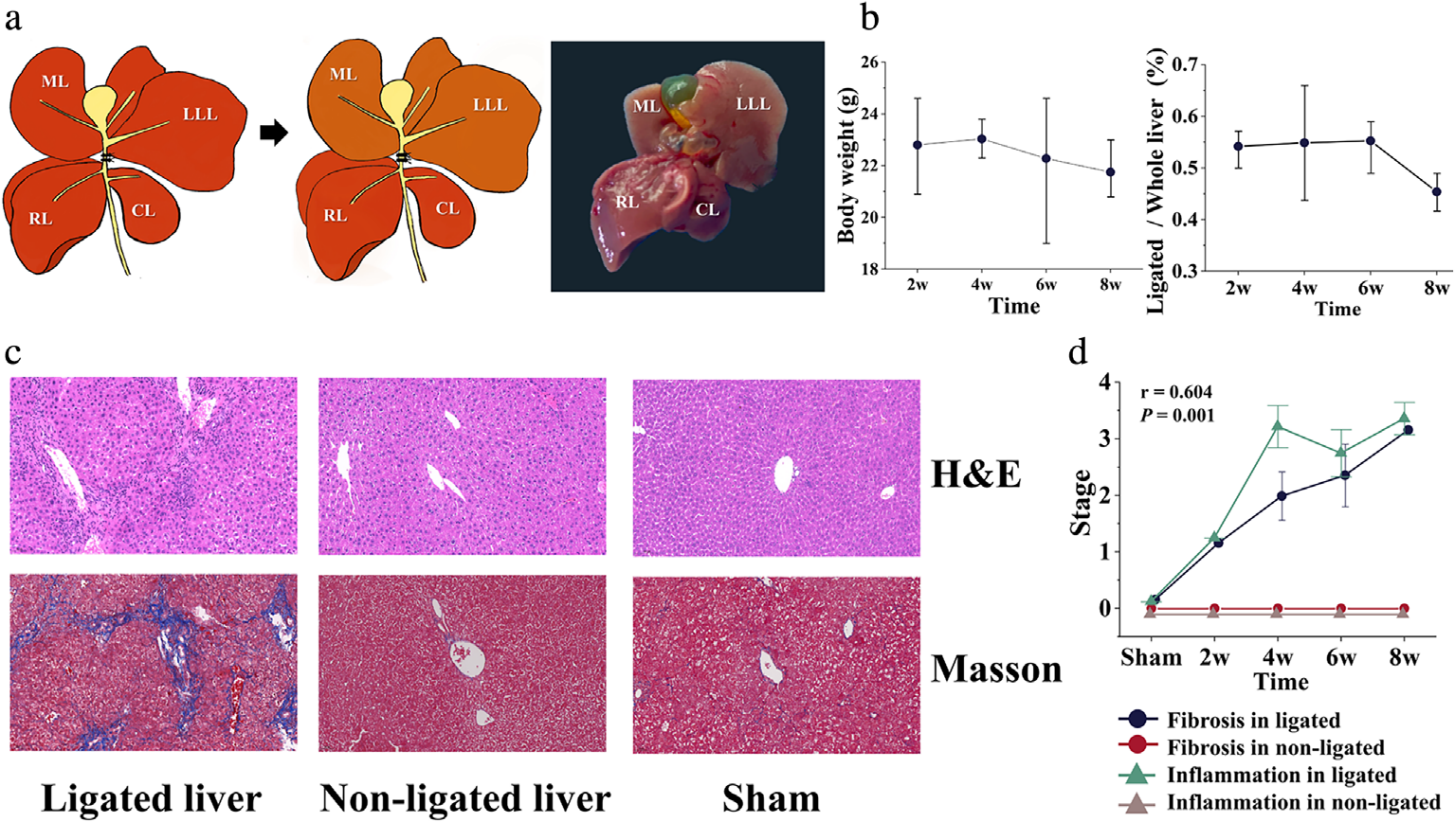
Images of PBDL model and pathological analysis. (**a**) Schematic diagram and image of the PBDL model. After ligating the confluence of the left and median bile duct, the left and median lobes gradually developed liver fibrosis. (**b**) Changes in mouse body weight (g) and the ratio of ligated liver/whole liver weight (%) during surgery in the PBDL model. (**c**) H&E and Masson staining of the liver in the 8‐week PBDL and sham groups (light microscopy, ×200). (**d**) Distribution of fibrosis and inflammation score stage in PBDL subgroups and sham group. PBDL = partial bile duct ligation; ML = median lobe; LLL = left lateral lobe; RL = right lobe; CL = caudated lobe.

After the sterilization of the surgical instruments and surgical table, the mouse that was meant to undergo surgery was anesthetized by intraperitoneal injection of 1% pentobarbital sodium solution (50 mg/kg). Thereafter, the mouse was fixed on the surgical table, and the abdominal fur was shaved. Isoflurane inhalation was used to maintain general anesthesia. For PBDL surgery, a long middle abdominal incision from the xiphoid to the pubis was made, and a moistened cotton swab was used to retract the liver towards the head to expose the hepatic hilum region. After confirming the location of the confluence of the left and median bile duct, a 5–0 suture was placed underneath the confluence and secured with two surgical knots (Fig 1a). Finally, the abdominal layers were closed with 6–0 absorbable sutures. For sham surgery, the abdominal wall was opened with the same middle incision and subsequently sutured.

### MRI Acquisition

MRI examinations were performed using a 3.0‐T clinical MR scanner (uMR 790, United Imaging Healthcare, Shanghai, China) equipped with 8‐channel mouse coil. Mice were anesthetized with 1% pentobarbital sodium solution (50 mg/kg) intraperitoneal injection before scan placed in the head‐first, prone position. The chest and abdomen of the mice were fixed with a manual bandage made of medical tape to decrease respiratory motion artifacts.

First, unenhanced transverse sequences of the whole liver were obtained including: T1‐weighted fast spin‐echo (FSE), T2‐weighted single shot FSE, diffusion‐weighted imaging (DWI) with nine *b* values (*b* = 0, 50, 100, 150, 200, 400, 600, 800, 1000 s/mm^2^), variable flip angle T1 mapping, multi‐echo spin‐echo T2 mapping, and multi‐echo gradient‐echo T2* mapping. After initial image acquisition, a dose of 0.02 mL Magnevist (Gadopentetate dimeglumine, Bayer Healthcare, Bayer) diluted with 0.08 mL saline was injected. Then, an additional set of T1‐weighted images (immediately after injection) and T1 mapping (5 minutes after injection) were acquired. All detailed parameters were listed in Table 1.

**TABLE 1:** Imaging Protocol for Magnetic Resonance Imaging (MRI) Sequences

Parameters/Sequences	T1WI	T2WI	EPI‐DWI	T1 Map	T2 Map	T2* Map
TR (msec)	585	2298	2000	15	3000	694.7
TE (msec)	12.54	84.48	80	3.87	17.4, 34.8, 52.2, 69.6, 87.0, 104.4	6.71, 17.89, 29.07, 40.25, 51.43
Flip angle (°)	150°	140°	90°	5°, 26°	180°	60°
Voxel size (mm^3^) *x* × *y* × *z*	0.16 × 0.16 × 1	0.20 × 0.20 × 2	0.59 × 0.59 × 2	0.13 × 0.13 × 1	0.50 × 0.50 × 2	0.38 × 0.38 × 2
FOV (mm^2^)	35 × 35	40 × 40	75 × 75	75 × 83	95 × 96	120 × 120
Slices	20	20	15	20	15	10
Acquisition time	4:12	5:25	3:22	1:41	6:06	2:00

T1WI = T1‐weighted imaging; T2WI = T2‐weighted imaging; EPI = echo‐planar imaging; DWI = diffusion‐weighted imaging; TR = repetition time; TE = echo time; FOV = field of view.

### Image Analysis

The T2 and T2* maps were generated using a commercial workstation (uWS‐MR, United Imaging Healthcare, Shanghai, China), which uses a log‐linear, least squares method to fit the echo intensities pixel‐by‐pixel, where the first echo is discarded due to the short TE effect on the T2 calculation.^16^, ^17^ Pixel‐wise T1 maps were generated using the Levenberg–Marquardt curve fitting algorithm on the uWS‐MR workstation.

Regions of interest (ROI) within ligated and non‐ligated liver in PBDL model and within whole liver in sham model were duplicated in the corresponding sequences. Then these ROIs were revised by the conventional T1 or T2 weighted images and drawn by an expert radiologist (Y‐HL, a 15‐year experience of abdomen imaging diagnosis) to calculate the T2 value, T2* value, native T1 value, and post‐contrast T1 value; vessels and bile duct were excluded. △T1 value was calculated as △T1 = T1_native_ − T1_post_.

For each mouse, DWI analysis was performed by an in‐house prototype software developed in MATLAB R2018b (MathWorks, Natick, MA). Apparent diffusion coefficient (ADC) was calculated voxel‐wise using two *b*‐values = 0 and 800 s/mm^2^, as defined in Eq. 1

(1) $$\frac{S_{b}}{S_{0}}=e^{-b\mathrm{ADC}}$$

where *S*_b_ and *S*_0_ denote the signal intensity with and without diffusion weighting, and *b* is the diffusion‐sensitizing factor.

Intravoxel incoherent motion (IVIM) parameter estimation was performed using all the nine *b* values (0–1000 s/mm^2^). In the bi‐exponential IVIM model, signal behavior is as follows:

(2) $$\frac{S_{b}}{S_{0}}=\left(1-f\right)\times \exp\left(-b\times D\right)+f\times \exp\left(-b\times D^{*}\right)$$

where *f* is the fractional perfusion related to microcirculation, *D* is the true diffusion as reflected by pure molecular diffusion, and D* is the pseudo‐diffusion coefficient related to perfusion. ROIs were manually drawn in the ligated and non‐ligated liver lobes in PBDL model and in whole segments in sham model (excluding the visible vessels and bile ducts).

ADC map data were loaded into the Parametric MRI application^18^ (https://www.parametricmri.com, Philadelphia, PA, USA) for histogram analysis. On the ADC map, ROIs were drawn at the boundary of the ligated and non‐ligated liver lobes in PBDL mouse and the boundary of whole liver in sham mouse. All measurements were performed by a radiologist (Y‐HL). Histogram parameters kurtosis, skewness, and ADC (mean) were calculated and analyzed.

### Histological Analysis

All mice were killed after MRI examination at their corresponding timepoints. The ligated and non‐ligated liver lobes from the PBDL model and the whole liver from the sham model were harvested and fixed in formalin independently for 48 hours before paraffin embedding.

Transverse sections were cut and stained with hematoxylin and eosin (H&E) and Masson's trichrome (G1006, Servicebio, Wuhan, China) to detect and stage fibrosis and inflammation. Images were observed under an Olympus microscope (DP72, Olympus Corporation, Tokyo, Japan) and captured by a camera (NIKON digital sight DS‐FI2) at a resolution of 1280*960. A pathologist (LL) with 10 years of experience who was blinded to the study performed the analysis. Fibrosis and inflammation were assessed and staged according to the Metavir and activity scoring systems.^19^, ^20^ Fibrosis was staged as follows. F0: no fibrosis; F1: periportal fibrosis; F2: rare fibrosis; F3: severe fibrosis without cirrhosis; and F4: cirrhosis. Inflammation was staged as follows. A0: no activity; A1: mild activity; A2: moderate activity; and A3: severe activity.

### Statistical Analysis

To determine the differences in parameters between the ligated, non‐ligated, and sham groups, one‐way ANOVA was performed in normally distributed parameters, and Kruskal–Wallis test was performed in non‐normally distributed parameters. The association between MRI parameters and histological characteristics was assessed using the Spearman's rank correlation. To characterize the relationship between fibrosis and inflammation in liver fibrosis, Spearman's rank correlation analysis was also performed between the fibrosis stage and inflammation stage in the ligated livers over time. After forward selection of fibrosis relative MR parameters with *r* > 0.60 and *P* < 0.05 at fibrosis stage correlation analysis, the receiver operating characteristic (ROC) curve for each parameter and for a combination of all parameters were performed to determine their capability in diagnosing liver fibrosis. Area under the curve (AUC), sensitivity, specificity, and cut‐off value were calculated. The Youden index was used to determine the optimal cut‐off value. All statistical analyses were performed using SPSS 22.0 (SPSS Inc., Chicago, IL, USA) and OriginPro 2021 (OriginLab, Northampton, MA, USA). *P*‐values were corrected using Bonferroni correction, and a *P‐*value <0.05 was considered statistically significant.

## Results

### Animal Model

No death was observed in both PBDL and sham groups during the study. The mice began losing weight postoperatively when measured either by body weight (almost 5% body weight loss at the end of 8 weeks after surgery) or the ligated/whole liver weight ratio (almost 17% decreased at the end of 8 weeks after surgery), Fig. 1b showed the general downward trends (*P* = 0.715 and *P* = 0.259).

### Changes in T1, T2, and T2* Mappings

**FIGURE 2:**
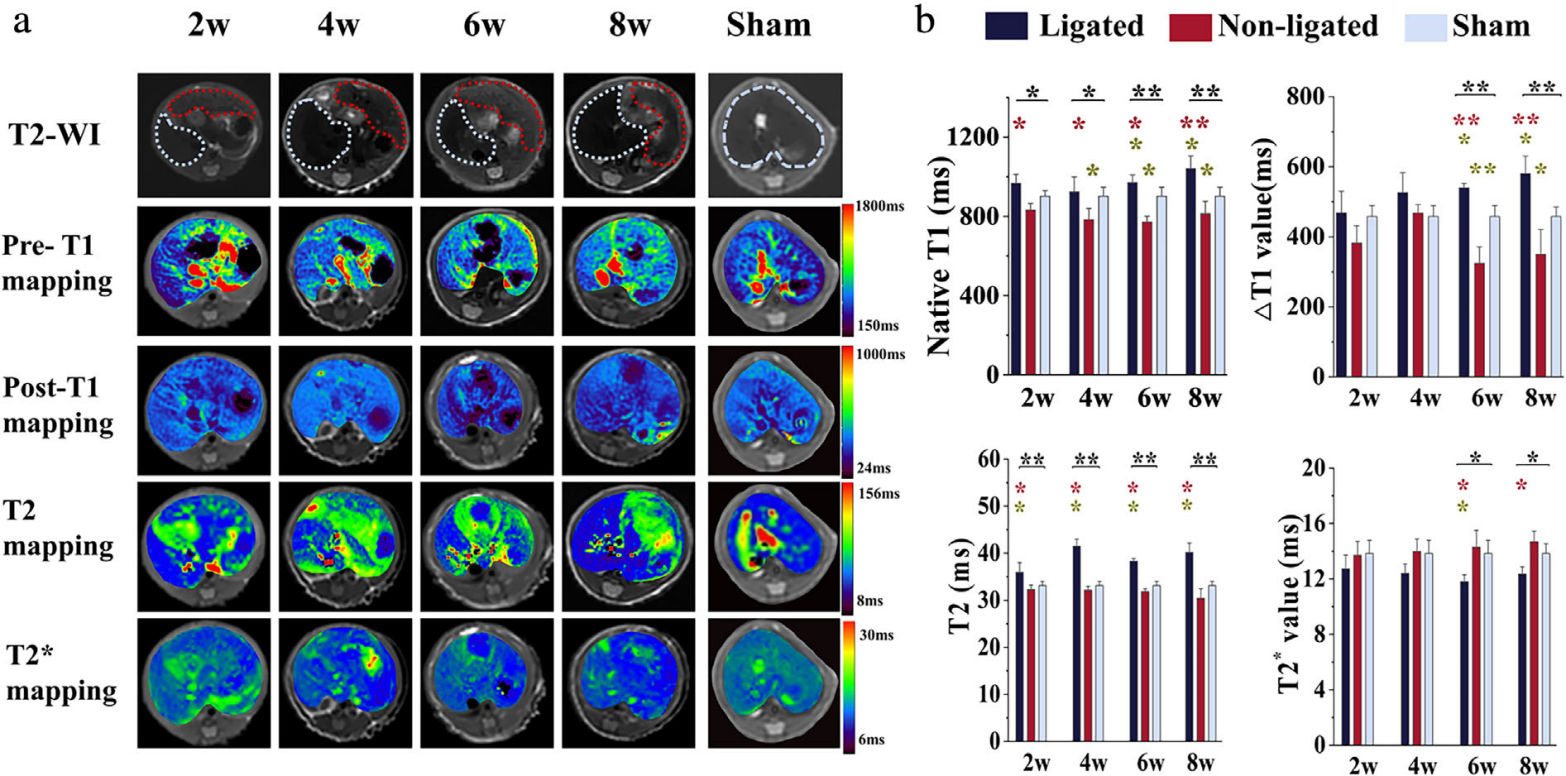
Images and plots of MRI parameter changes in the PBDL and sham liver groups. (**a**) T2‐weighted images, pre‐contrast T1 maps, post‐contrast T1 maps, T2 maps, and T2^*^ maps in the four timepoints for PBDL and sham groups. Dotted lines in T2‐WI images are shown as the boundaries of the ligated (red) and non‐ligated (blue) liver lobes in the PBDL group and the whole liver (blue) in the sham group. (**b**) Bar chart of native T1, △T1, T2, and T2^*^ values. *P < 0.05, **P < 0.001 (black) for comparison between the ligated, non‐ligated and sham liver. *P < 0.05, **P < 0.001 (red) indicate the significance when compared to the non‐ligated liver. *P < 0.05, **P < 0.001 (yellow) indicate the significance when compared to the sham liver. PBDL = partial bile duct ligation; T2‐WI = T2‐weighted images.

Pre‐contrast T1 maps and post‐contrast T1 maps, T2, and T2* maps of the PBDL model at four timepoints of analysis and of the sham group are shown in Fig. 2 and Table 2. At all four timepoints, the native T1 and T2 values were significantly higher in the ligated livers than in non‐ligated or sham livers. △T1 values were significantly higher in the ligated liver model (in the 6‐week and 8‐week groups) than in the non‐ligated liver and sham groups (*P* = 0.068 in 2 weeks group; *P* = 0.135 in 4 weeks group). T2^*^ values were significantly lower in the ligated livers in 6‐week and 8‐week groups than other livers (*P* = 0.175 in 2 weeks group; *P* = 0.067 in 4 weeks group).

**TABLE 2:** Comparison of Significant MR Parameters Between Ligated, Non‐Ligated and Sham Livers

Parameters	2 Weeks				4 Weeks				6 Weeks				8 Weeks			
	Ligated	Non‐Ligated	Sham	*P*‐Value	Ligated	Non‐Ligated	Sham	*P*‐Value	Ligated	Non‐Ligated	Sham	*P*‐Value	Ligated	Non‐Ligated	Sham	*P*‐Value
Native T1 (msec)	966.31 ± 125.350	832.903 ± 77.624	901.000 ± 58.541	0.007*	923.000 ± 90.563	783.938 ± 67.704	901.000 ± 58.541	0.003*	970.500 ± 49.003	771.703 ± 37.172	901.000 ± 58.541	<0.001**	1040.174 ± 69.728	814.143 ± 66.827	901.000 ± 58.541	<0.001**
ΔT1 (msec)	468.083 ± 91.293	382.190 ± 73.450	458.375 ± 40.281	0.068	525.875 ± 85.271	467.900 ± 36.292	458.375 ± 40.281	0.135	539.750 ± 18.321	324.533 ± 69.037	458.375 ± 40.281	<0.001**	580.176 ± 74.484	349.867 ± 105.714	458.375 ± 40.281	<0.001**
T2 (msec)	35.922 ± 2.652	32.239 ± 1.426	33.087 ± 1.489	<0.001**	41.49 ± 2.227	32.136 ± 0.877	33.087 ± 1.489	<0.001**	38.312 ± 0.666	31.864 ± 0.597	33.087 ± 1.489	<0.001**	40.145 ± 2.932	30.429 ± 2.413	33.087 ± 1.489	<0.001**
T2* (msec)	12.729 ± 1.695	13.705 ± 1.727	13.833 ± 1.235	0.175	12.405 ± 1.172	13.981 ± 1.570	13.833 ± 1.235	0.067	11.794 ± 0.869	14.293 ± 0.972	13.833 ± 1.235	0.002*	12.355 ± 0.910	14.683 ± 1.327	13.833 ± 1.235	0.017*
ADC (×10^−3^, mm^2^/s)	1.179 ± 0.108	1.292 ± 0.129	1.363 ± 0.057	0.016*	1.258 ± 0.059	1.370 ± 0.103	1.363 ± 0.057	0.085	1.179 ± 0.104	1.292 ± 0.122	1.363 ± 0.057	0.075	1.097 ± 0.065	1.360 ± 0.229	1.363 ± 0.057	0.048*
*D* (×10^−3^, mm^2^/s)	1.087 ± 0.129	1.009 ± 0.115	1.021 ± 0.100	0.321	0.915 ± 0.092	1.001 ± 0.251	1.021 ± 0.100	0.486	0.869 ± 0.164	0.930 ± 0.158	1.021 ± 0.100	0.206	0.959 ± 0.073	0.946 ± 0.0690	1.021 ± 0.100	0.395

*P*‐value represented the comparison between three groups.

* *P* < 0.05.

** *P* < 0.001.

Regarding to the comparison between the non‐ligated and sham liver, only native T1 value (in 4, 6, and 8 weeks) and △T1 value (in 6 and 8 weeks) within the non‐ligated liver showed significant decreases. Native T1 in 2 weeks (*P* = 0.284), △T1 in 2 weeks (*P* = 0.093) and 4 weeks (*P* = 0.946), T2 in four timepoints (*P* = 0.512 in 2 weeks, *P* = 0.466 in 4 weeks, *P* = 0.098 in 6 weeks, and *P* = 0.310 in 8 weeks) and T2^*^ in four timepoints (*P* = 1.000 in 2 weeks, *P* = 0.974 in 4 weeks, *P* = 0.729 in 6 weeks, and *P* = 0.461 in 8 weeks) within the non‐ligated liver showed no significant differences with the sham liver.

### Changes in DWI and IVIM

**FIGURE 3:**
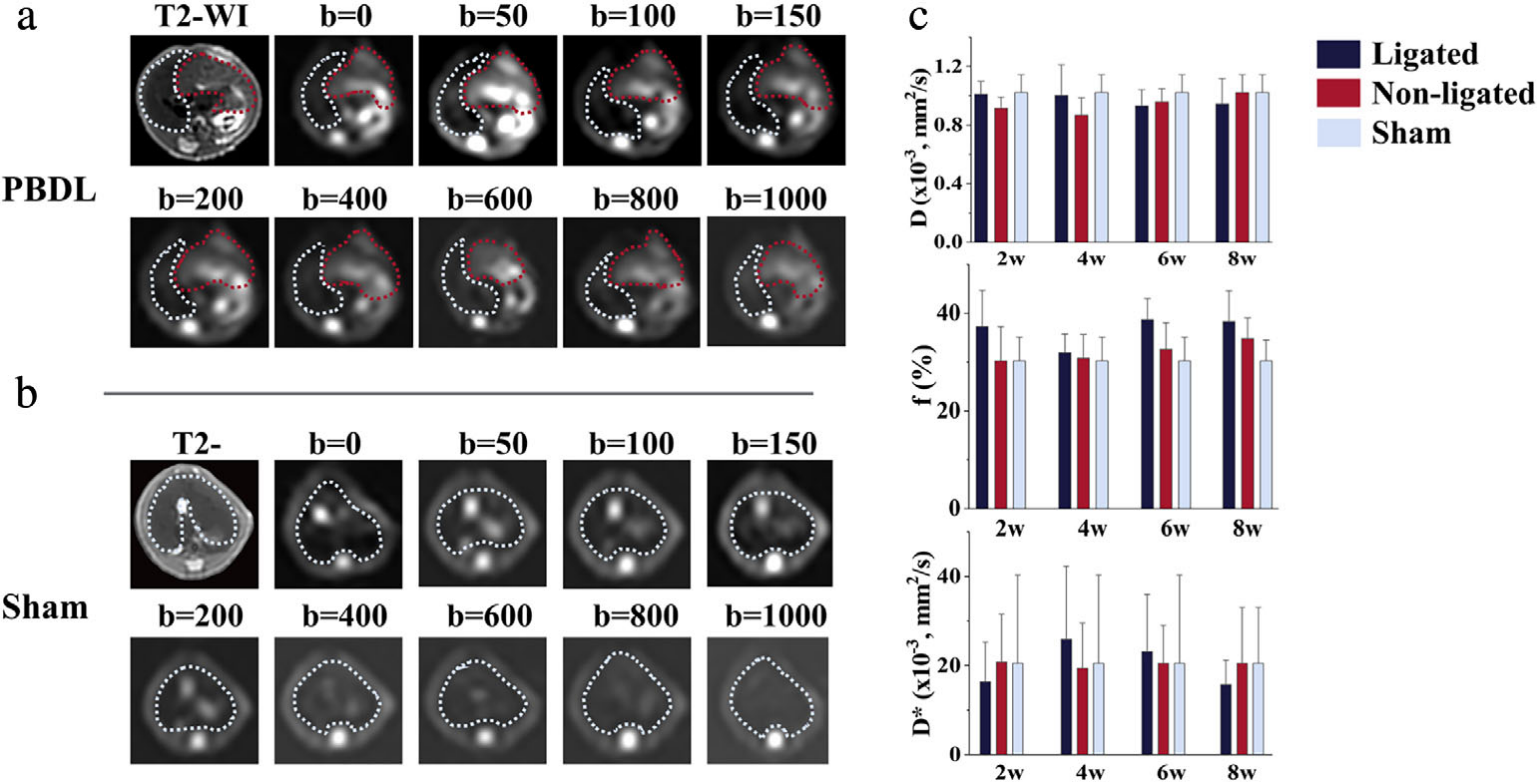
Images and diagrams of IVIM parameter changes in the PBDL and sham groups. (**a**) Nine *b*‐value images of PBDL and sham livers, which were used to calculate IVIM parameters. In the PBDL liver group, the ligated (red) and non‐ligated (blue) lobes were distinguished by the dotted line, the boundaries of the whole liver are also shown as a blue dotted line. (**b**) Bar chart of *f*, *D*, and D* of the ligated, non‐ligated, and sham livers. *P < 0.05, **P < 0.001 (black) for comparison between the ligated, non‐ligated and sham liver. *P < 0.05, **P < 0.001 (red) indicate the significance when compared to the non‐ligated liver. *P < 0.05, **P < 0.001 (yellow) indicate the significance when compared to the sham liver. PBDL = partial bile duct ligation; IVIM = intravoxel incoherent motion.

**FIGURE 4:**
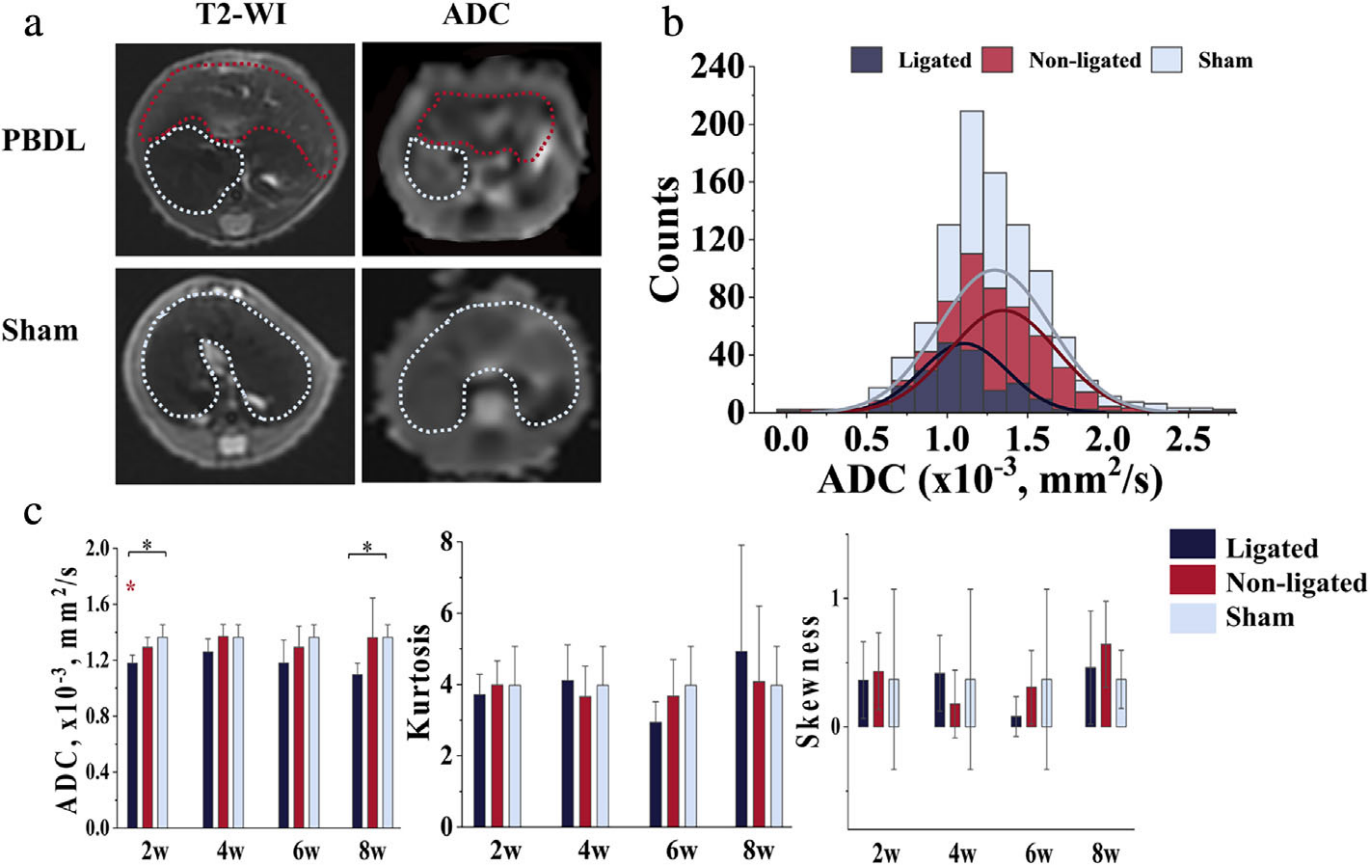
Images and diagrams of ADC histogram analysis. (**a**) T2‐weight images and ADC images of PBDL and sham livers. In PBDL livers, the ligated (red) and non‐ligated (blue) lobes were distinguished by the dotted line, the boundaries of the whole liver are also shown as a blue dotted line. (**b**) Histograms of the ligated, non‐ligated, and sham livers. (**c**) Bar chart of ADC and Kurtosis and box chart of Skewness. *P < 0.05, **P < 0.001 (black) for comparison between the ligated, non‐ligated and sham liver. *P < 0.05, **P < 0.001 (red) indicate the significance when compared to the non‐ligated liver. *P < 0.05, **P < 0.001 (yellow) indicate the significance when compared to the sham liver. PBDL: partial bile duct ligation; ADC = apparent diffusion coefficient.

Figure 3 shows the nine *b*‐value images that were used to calculate the IVIM parameters *D*, *D* ^*^, and *f*. The *D* in the ligated liver showed a slightly decreased trend compared to the non‐ligated and sham liver without significances in all four timepoints (*P* = 0.321 in 2 weeks, *P* = 0.486 in 4 weeks, *P* = 0.206 in 6 weeks, and *P* = 0.395 in 8 weeks). Although the *f* slightly increased in mice with ligated livers when compared to other livers, there were non‐significant differences between the ligated, non‐ligated and sham liver in all four timepoints (*P* = 0.273 in 2 weeks, *P* = 0.900 in 4 weeks, *P* = 0.087 in 6 weeks, and *P* = 0.253 in 8 weeks). *D* ^*^ also showed no statistical differences between ligated, non‐ligated, and sham liver groups (*P* = 0.677 in 2 weeks, *P* = 0.925 in 4 weeks, *P* = 0.806 in 6 weeks, and *P* = 0.993 in 8 weeks). An ADC map (shown in Fig. 4a) for each mouse was used to perform a histogram analysis (Fig. 4b). ADC histogram parameters are summarized in Fig. 4c. The ADC value decreased in the ligated livers with significance in 2‐week and 8‐week groups (*P* = 0.057 in 4 weeks and *P* = 0.075 in 6 weeks). There were no statistically significant differences in skewness (*P* = 0.620 in 2 weeks, *P* = 0.757 in 4 weeks, *P* = 0.113 in 6 weeks, and *P* = 0.777 in 8 weeks) and kurtosis (*P* = 0.721 in 2 weeks, *P* = 0.703 in 4 weeks, *P* = 0.189 in 6 weeks, and *P* = 0.703 in 8 weeks) between three groups throughout all four timepoints.

### Histopathologic Analysis

Histological analysis showed that fibrosis development in the ligated liver model maintained a linear trend with significance (Fig. 1d) and was eventually staged as F3 at the end of 8 weeks after surgery (Fig. 1c, Masson). Figure 1d also presents inflammatory activity over time. Inflammatory activity gradually developed after ligation and ranked top (A3 stage) at 4 weeks after surgery except for a few decreases at 6 weeks (Fig. 1c, H&E). For the non‐ligated liver and sham groups, neither fibrosis nor active inflammation was observed. According to Spearman's correlation analysis, in cholestatic liver fibrosis process, fibrosis and inflammation had a moderate relationship (*r* = 0.604).

### Correlation Analysis

**FIGURE 5:**
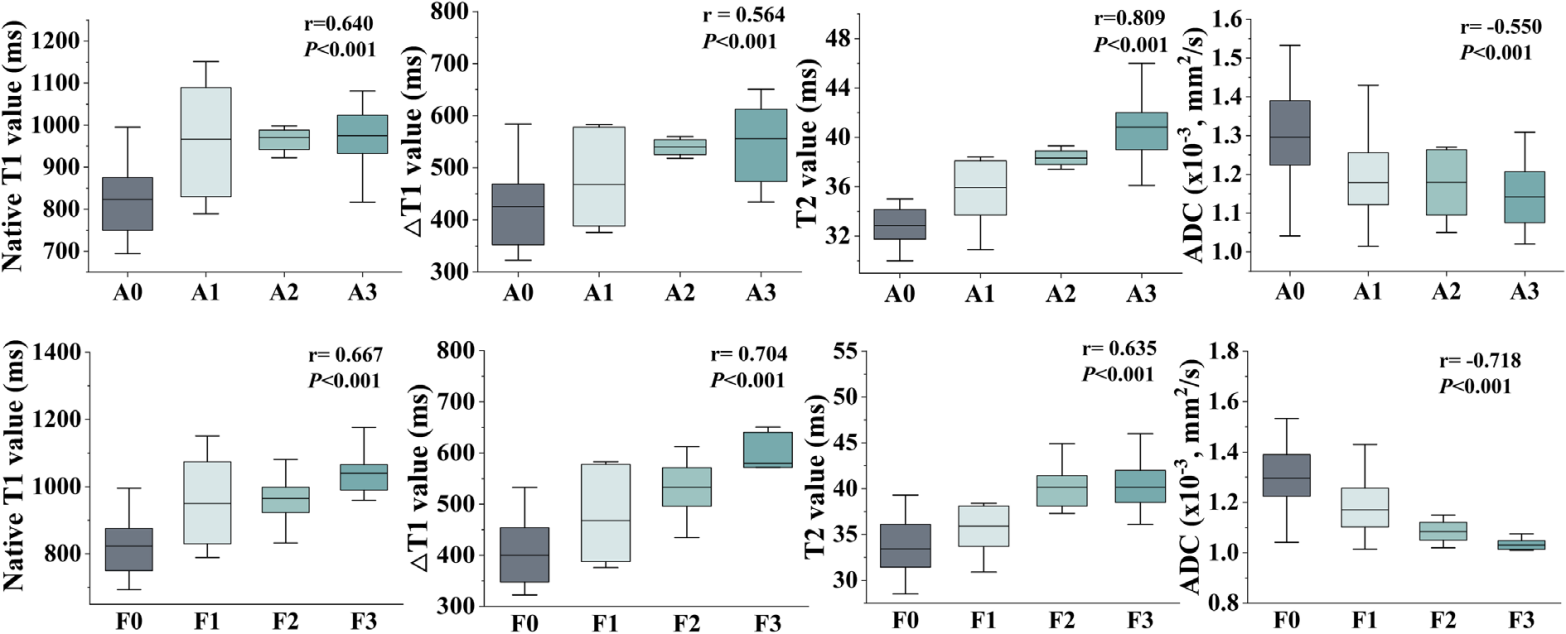
Spearman correlation between magnetic resonance imaging (MRI) parameters and fibrosis and inflammation.

As presented in Fig. 5 and Table 3, T2 values showed a strong correlation with inflammation (*r* = 0.809) and a moderate correlation with fibrosis (*r* = 0.635). △T1 better correlated with fibrosis (*r* = 0.704) than with inflammation (*r* = 0.564). The same result was also observed in ADC values (fibrosis: *r* = −0.718; inflammation: *r* = −0.550). A moderate positive correlation was observed between native T1 values and fibrosis (*r* = 0.667) and inflammation (*r* = 0.640). Other parameters that showed no or a weak correlation with fibrosis or inflammation are summarized in Fig. S1 in the Supplemental Material and Table 3. T2^*^ and *D* showed a weak negative correlation with both fibrosis (*r* = −0.411; *r* = −0.400, respectively) and inflammation (*r* = −0.472; *r* = −0.443, respectively). In addition, differences of MR parameters between 4 timepoints in the ligated liver were compared and shown in Fig. S2. According to the results, native T1, △T1, T2, ADC and *D* showed significance between 4 timepoints.

**TABLE 3:** Correlation Matrix for Magnetic Resonance Imaging (MRI) Parameters and Histological Features of Liver Fibrosis

Parameters	Fibrosis		Inflammation	
	*r* Value	*P*‐Value	*r* Value	*P*‐Value
Native T1 value	0.667	<0.001	0.640	<0.001
ΔT1 value	0.704	<0.001	0.564	<0.001
T2 value	0.635	<0.001	0.809	<0.001
T2^*^ value	−0.411	0.014	−0.472	0.003
ADC mean	−0.718	<0.001	−0.550	0.001
Skewness	−0.131	0.293	−0.235	0.033
Kurtosis	0.005	0.855	−0.084	0.435
*f*	0.105	0.420	0.071	0.585
*D* value	−0.400	0.002	−0.443	<0.001
*D* ^*^ value	−0.112	0.245	−0.095	0.361

### ROC Analysis

**FIGURE 6:**
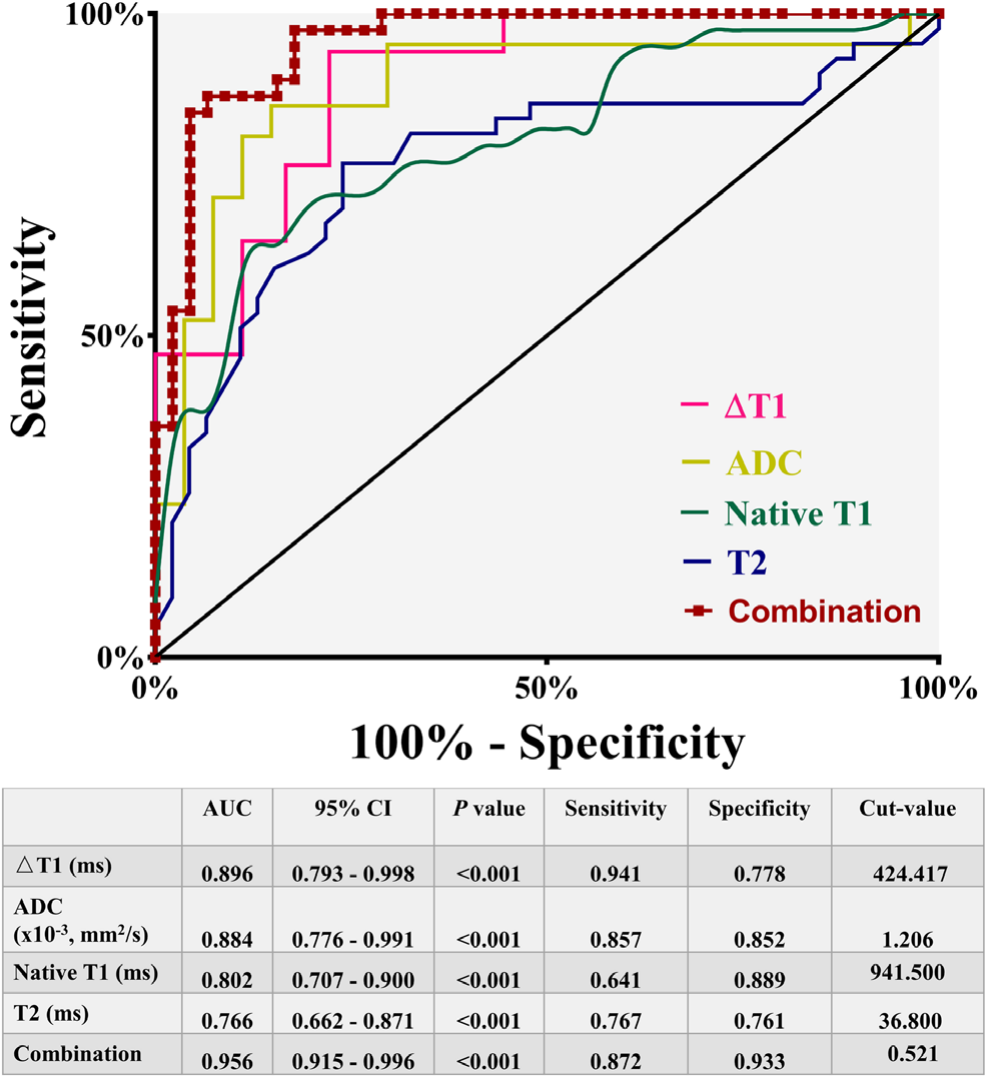
Receiver operating characteristic (ROC) curve for MR parameters (native T1, △T1, T2, and ADC), as well as the combination of all parameters. MR = magnetic resonance; ADC = apparent diffusion coefficient.

To determine the potential of diagnosing liver fibrosis using MRI parameters, ROC analysis was conducted for native T1, △T1, T2, and ADC. The results are shown in Fig. 6. Single parameter results indicated that AUC was the highest for the △T1 value (0.896), with a sensitivity of 0.941 and a specificity of 0.778. △T1, T2, and ADC were entered into joint detection equation (combination) for ROC analysis (also shown in Fig. 6). The analysis indicated that the AUC for the combination (0.956) was greater than that of any single parameter, with a sensitivity of 0.872 and a specificity of 0.933.

## Discussion

The results of this study demonstrated that the PBDL model is a reproducible model. Moreover, clinical 3.0 T MRI was satisfactory and demonstrated a strong correlation between MRI parameters and histopathological characteristics. These significant relationships can help to non‐invasively differentiate the level of inflammation and liver fibrosis in the preclinical studies.

The widely used animal models of liver fibrosis were mainly two types including hepatotoxic substance induction and surgical ligation, such as CCL4, TAA, DDC, and CBDL. At present, the CBDL model is the most widely used animal model in the cholestasis study, due to the simple procedure. However, the high mortality rate of the CBDL model is a non‐negligible weakness.^21^ Finally, we successfully established a PBDL model by strictly following the experimental protocol and precise operation. No death was observed in both PBDL and sham groups during the study. Meanwhile, we used retro‐orbital injection to further increase the reproducibility of the PBDL model because it has more repeated times of administrations and less impairment compared with tail vein injection.^22^

MRI features of cholestatic liver fibrosis in PBDL have not been well characterized. In addition, the previous studies of liver fibrosis mainly used rats or rabbits. But the results of this study demonstrate that the PBDL mouse model can be successfully characterized using MRI assessments with a satisfactory image quality. On the one hand, the volume of swollen bile ducts in the PBDL model was small in MR images, which is effective in reducing the squeeze and morphologic changes of adjacent organs. More detailed anatomical and functional information could not be covered. On the other hand, although low field‐strength clinical MRI was less powerful than specific small‐animal MRI, it proved to be advantageous for the easier translation of results to clinical human studies. In this study, we strenuously overcame the influences of respiratory motion artifacts via the manual bandage and reasonable anesthetic protocol and increased the number of excitations for image acquisition within the acceptable time range. In future investigations, the lack of an advanced animal MRI machine will not be an obstacle for performing scientific research on fibrosis in preclinical stages.

In our histological findings, we found that the formation of fibrosis and the activation of inflammation occur almost simultaneously in the ligated live lobes. Surprisingly, the non‐ligated liver lobes did not show significant inflammation activation and fibrosis formation. Therefore, PBDL as a self‐comparison model could be better to avoid the selection bias of different individuals. Meanwhile, two different pathological changes could be observed on the same transversal MR images, which will be beneficial to avoid the observation bias.

To explore MRI findings, a series of quantitative MRI sequences, including T1 mapping, T2 mapping, T2* mapping, IVIM, DWI, and contrast‐enhanced imaging, was used in this study. T1 and T2 mappings enabled the measurement of T1 and T2 values via the corresponding values in each voxel. Pre‐contrast T1 values which measured in the absence of a contrast agent reflect the signal changes in intracellular and extracellular compartments including collagen, water, and protein.^23^ Several reports have demonstrated the relationship between T1 values and fibrosis in both animals and humans.^24^ These results reckoned that pre‐contrast T1 values could be a noninvasive imaging tool for the diagnosis of liver fibrosis, even for differentiating early stages of fibrosis from normal liver.^25^, ^26^, ^27^ Although we also found a moderate correlation between T1 values and fibrosis, T1 value is not the best parameter in this study. Similarly, T2 values have also been demonstrated to calculate hepatic fibrosis, indicating a close relationship between increased T2 values and areas that develop fibrosis.^28^ Recently, researchers have shown an increased interest in the role of iron in liver fibrosis and suspect that it might facilitate fibrotic responses.^29^, ^30^ T2* mapping with a multi‐gradient echo technique has been recommended to assess iron overload.^31^ Post‐contrast T1 values reflect changes in hemodynamics.^32^ Compared with the other MRI parameters, △T1 value has the highest correlation with liver fibrosis. However, inflammation is the other important and unavoidable factor in liver fibrosis pathogenesis.^1^ Notably, the T2 value has the highest correlation with inflammation, and its correlation coefficient was higher than the remaining MR parameters.

DW imaging can detect the motion of water molecules within tissue voxels.^33^ IVIM imaging can assess the diffusion of water molecules and microcirculation perfusion from DWI datasets.^34^ In our study, this result may indicate that the ADC value may be a more promising parameter for evaluating fibrosis and not inflammation. Moreover, this is consistent with previous studies, which have shown that ADC values help assess liver fibrosis staging not only in animals but also in humans^35^, ^36^ and that IVIM imaging is weakly correlated with inflammation.^37^ D* and *f* values represent perfusion in biological tissues.^38^ A possible explanation for this might be that microcirculation perfusion is not affected in PBDL models.^6^ Moreover, kurtosis and skewness reflect the peak and asymmetric distribution of the ADC histogram.^39^ There were no differences including D*, *f*, kurtosis, and skewness between the groups in this study; therefore, it was difficult to ascertain how these parameters as mentioned above reflect inflammation or fibrosis.

## Limitations

Firstly, we attempted to match the ROIs placed in the multiple MRI sequences; however, an exact match could not be achieved due to the partial volume effect from the different thicknesses of MRI images. Secondly, there are bile duct mutations in the PBDL model, which would give us some challenges for surgical procedures and MRI evaluation. Fortunately, through our experiments, we found that bile duct variation is rare when ligating the intersection of the left lobe and the middle lobe of the common bile duct. Thirdly, while the deformation of DWI and IVIM was minimal, this was also a possible limitation because of their insensitivity to movement. Although our results from clinical MRI were satisfactory and were easier to realize for clinical translation, the optimization of MRI sequence and higher magnetic field will help to display more image information for further investigations.

## Conclusions

PBDL is a high reproducible mouse model to mimic the formation of secondary biliary fibrosis. Multiparametric MRI is a reliable tool for the in vivo noninvasive characterization of the PBDL model. The simultaneous presence of inflammation and fibrosis were differentiated by T2 values, ADC, and △T1, separately, which provided new insight into the differentiation of overlapped histopathological findings in MR images. Compared with the other MRI parameters, △T1 and ADC values have a higher correlation with liver fibrosis, while T2 values have a higher correlation with inflammation.

## Conflict of Interest

The authors declare no potential conflicts of interest.

## Supporting information


**Fig S1** Spearman correlation between other MRI parameters and fibrosis and inflammation.

**Fig S2** Comparison of all MR parameters between the four different timepoints in the ligated liver group. *P < 0.05 and **P < 0.01.

## References

[1] Kisseleva T , Brenner D . Molecular and cellular mechanisms of liver fibrosis and its regression. Nat Rev Gastroenterol Hepatol 2021;18:151‐166. 10.1038/s41575-020-00372-7. 33128017

[2] Han H , Desert R , Das S , et al. Danger signals in liver injury and restoration of homeostasis. J Hepatol 2020;73:933‐951. 10.1016/j.jhep.2020.04.033. 32371195 PMC7502511

[3] Schuppan D , Afdhal NH . Liver cirrhosis. Lancet (London, England) 2008;371:838‐851. 10.1016/S0140-6736(08)60383-9. PMC2271178 18328931

[4] Guillot A , Guerri L , Feng D , et al. Bile acid‐activated macrophages promote biliary epithelial cell proliferation through integrin αvβ6 upregulation following liver injury. J Clin Invest 2021;131:e132305. 10.1172/jci132305. PMC8087210 33724957

[5] D'Mello C , Almishri W , Liu H , Swain M . Interactions between platelets and inflammatory monocytes affect sickness behavior in mice with liver inflammation. Gastroenterology 2017;153:1416‐1428. 10.1053/j.gastro.2017.08.011. 28802564

[6] Yokota S , Ono Y , Nakao T , Zhang P , Michalopoulos GK , Khan Z . Partial bile duct ligation in the mouse: A controlled model of localized obstructive cholestasis. J Vis Exp 2018;133:56930. 10.3791/56930. PMC5933264 29658929

[7] Aske KC , Waugh CA . Expanding the 3R principles: More rigour and transparency in research using animals. EMBO Rep 2017;18:1490‐1492. 10.15252/embr.201744428. 28743713 PMC5579369

[8] Hurst J , West R . Taming anxiety in laboratory mice. Nat Methods 2010;7:825‐826. 10.1038/nmeth.1500. 20835246

[9] Liu J , Li Y , Zhang J , et al. Comparison of anesthesia and tumor implantation methods for establishing rabbit VX2 hepatocarcinoma. Am J Transl Res 2019;11:7157‐7165. 31814918 PMC6895537

[10] Preis E , Schulze J , Gutberlet B , Pinnapireddy SR , Jedelská J , Bakowsky U . The chorioallantoic membrane as a bio‐barrier model for the evaluation of nanoscale drug delivery systems for tumour therapy. Adv Drug Deliv Rev 2021;174:317‐336. 10.1016/j.addr.2021.04.022. 33905805

[11] Taoka T , Jost G , Frenzel T , Naganawa S , Pietsch H . Impact of the glymphatic system on the kinetic and distribution of gadodiamide in the rat brain: Observations by dynamic MRI and effect of circadian rhythm on tissue gadolinium concentrations. Invest Radiol 2018;53:529‐534. 10.1097/rli.0000000000000473. 29652699

[12] Scarfe L , Taylor A , Sharkey J , et al. Non‐invasive imaging reveals conditions that impact distribution and persistence of cells after in vivo administration. Stem Cell Res Ther 2018;9:332. 10.1186/s13287-018-1076-x. 30486897 PMC6264053

[13] Baier J , Rix A , Drude NI , et al. Influence of MRI examinations on animal welfare and study results. Invest Radiol 2020;55:507‐514. 10.1097/rli.0000000000000669. 32224718

[14] Allkemper T , Sagmeister F , Cicinnati V , et al. Evaluation of fibrotic liver disease with whole‐liver T1ρ MR imaging: A feasibility study at 1.5 T. Radiology 2014;271:408‐415. 10.1148/radiol.13130342. 24475807

[15] Pavlides M , Banerjee R , Sellwood J , et al. Multiparametric magnetic resonance imaging predicts clinical outcomes in patients with chronic liver disease. J Hepatol 2016;64:308‐315. 10.1016/j.jhep.2015.10.009. 26471505 PMC4751288

[16] Milford D , Rosbach N , Bendszus M , Heiland SJP . Mono‐exponential fitting in T2‐relaxometry: Relevance of offset and first echo. 2015;10:e0145255. 10.1371/journal.pone.0145255 PMC4683054 26678918

[17] Mamisch TC , Hughes T , Mosher TJ , et al. T2 star relaxation times for assessment of articular cartilage at 3 T: A feasibility study. 2012;41:287‐292. 10.1007/s00256-011-1171-x 21499976

[18] Calle‐Toro JS , Barrera CA , Khrichenko D , Otero HJ , Serai SD . R2 relaxometry based MR imaging for estimation of liver iron content: A comparison between two methods. Abdom Radiol (NY) 2019;44:3058‐3068. 10.1007/s00261-019-02074-4. 31161282

[19] Rousselet MC , Michalak S , Dupré F , et al. Sources of variability in histological scoring of chronic viral hepatitis. Hepatology 2005;41:257‐264. 10.1002/hep.20535. 15660389

[20] Bedossa P , Poitou C , Veyrie N , et al. Histopathological algorithm and scoring system for evaluation of liver lesions in morbidly obese patients. 2012;56:1751‐1759. 10.1002/hep.25889 22707395

[21] Nevzorova YA , Boyer‐Diaz Z , Cubero FJ , Gracia‐Sancho J . Animal models for liver disease – A practical approach for translational research. J Hepatol 2020;73:423‐440. 10.1016/j.jhep.2020.04.011. 32330604

[22] Yardeni T , Eckhaus M , Morris HD , Huizing M , Hoogstraten‐Miller S . Retro‐orbital injections in mice. Lab Anim (NY) 2011;40:155‐160. 10.1038/laban0511-155. 21508954 PMC3158461

[23] Haaf P , Garg P , Messroghli DR , Broadbent DA , Greenwood JP , Plein S . Cardiac T1 mapping and extracellular volume (ECV) in clinical practice: A comprehensive review. J Cardiovasc Magn Reson 2016;18:89. 10.1186/s12968-016-0308-4. 27899132 PMC5129251

[24] Taylor AJ , Salerno M , Dharmakumar R , Jerosch‐Herold M . T1 mapping: Basic techniques and clinical applications. J Am Coll Cardiol Img 2016;9:67‐81. 10.1016/j.jcmg.2015.11.005. 26762877

[25] Banerjee R , Pavlides M , Tunnicliffe EM , et al. Multiparametric magnetic resonance for the non‐invasive diagnosis of liver disease. J Hepatol 2014;60:69‐77. 10.1016/j.jhep.2013.09.002. 24036007 PMC3865797

[26] Li Z , Sun J , Hu X , et al. Assessment of liver fibrosis by variable flip angle T1 mapping at 3.0T. J Magn Reson Imaging 2016;43:698‐703. 10.1002/jmri.25030. 26267123

[27] Li J , Liu H , Zhang C , et al. Native T1 mapping compared to ultrasound elastography for staging and monitoring liver fibrosis: An animal study of repeatability, reproducibility, and accuracy. Eur Radiol 2020;30:337‐345. 10.1007/s00330-019-06335-0. 31338650

[28] Guimaraes AR , Siqueira L , Uppal R , et al. T2 relaxation time is related to liver fibrosis severity. Quant Imaging Med Surg 2016;6:103‐114. 10.21037/qims.2016.03.02. 27190762 PMC4858462

[29] Yu Y , Jiang L , Wang H , et al. Hepatic transferrin plays a role in systemic iron homeostasis and liver ferroptosis. Blood 2020;136:726‐739. 10.1182/blood.2019002907. 32374849 PMC7414596

[30] Preziosi ME , Singh S , Valore EV , et al. Mice lacking liver‐specific β‐catenin develop steatohepatitis and fibrosis after iron overload. J Hepatol 2017;67:360‐369. 10.1016/j.jhep.2017.03.012. 28341391 PMC5515705

[31] Bush AM , Sandino CM , Ramachandran S , et al. Rosette trajectories enable ungated, motion‐robust, simultaneous cardiac and liver T(2)* iron assessment. J Magn Reson Imaging 2020;52:1688‐1698. 10.1002/jmri.27196. 32452088 PMC7699670

[32] Wang B , Zhang Y , Zhao B , et al. Postcontrast T1 mapping for differential diagnosis of recurrence and radionecrosis after gamma knife radiosurgery for brain metastasis. Am J Neuroradiol 2018;39:1025‐1031. 10.3174/ajnr.A5643. 29724761 PMC7410634

[33] Qi J , Olsen NJ , Price RR , Winston JA , Park JH . Diffusion‐weighted imaging of inflammatory myopathies: Polymyositis and dermatomyositis. J Magn Reson Imaging 2008;27:212‐217. 10.1002/jmri.21209. 18022843

[34] Bergamino M , Nespodzany A , Baxter LC , et al. Preliminary assessment of intravoxel incoherent motion diffusion‐weighted MRI (IVIM‐DWI) metrics in Alzheimer's disease. J Magn Reson Imaging 2020;52:1811‐1826. 10.1002/jmri.27272. 32621405

[35] Hu G , Zhang X , Liang W , et al. Assessment of liver fibrosis in rats by MRI with apparent diffusion coefficient and T1 relaxation time in the rotating frame. J Magn Reson Imaging 2016;43:1082‐1089. 10.1002/jmri.25084. 26497954

[36] Kromrey M , Le Bihan D , Ichikawa S , Motosugi U . Diffusion‐weighted MRI‐based virtual elastography for the assessment of liver fibrosis. Radiology 2020;295:127‐135. 10.1148/radiol.2020191498. 32043948

[37] Lefebvre T , Hébert M , Bilodeau L , et al. Intravoxel incoherent motion diffusion‐weighted MRI for the characterization of inflammation in chronic liver disease. Eur Radiol 2021;31:1347‐1358. 10.1007/s00330-020-07203-y. 32876833

[38] Dijkstra H , Dorrius MD , Wielema M , Pijnappel RM , Oudkerk M , Sijens PE . Quantitative DWI implemented after DCE‐MRI yields increased specificity for BI‐RADS 3 and 4 breast lesions. J Magn Reson Imaging 2016;44:1642‐1649. 10.1002/jmri.25331. 27273694

[39] Just N . Improving tumour heterogeneity MRI assessment with histograms. Br J Cancer 2014;111:2205‐2213. 10.1038/bjc.2014.512. 25268373 PMC4264439

