# Supplementary figures and images for: Characterizing Fibrosis and Inflammation in a Partial Bile Duct Ligation Mouse Model by Multiparametric Magnetic Resonance Imaging
Source: J Magn Reson Imaging. 2021 Sep 21;55(6):1864–74. doi: 10.1002/jmri.27925 (PMC9290705; doi:10.1002/jmri.27925)

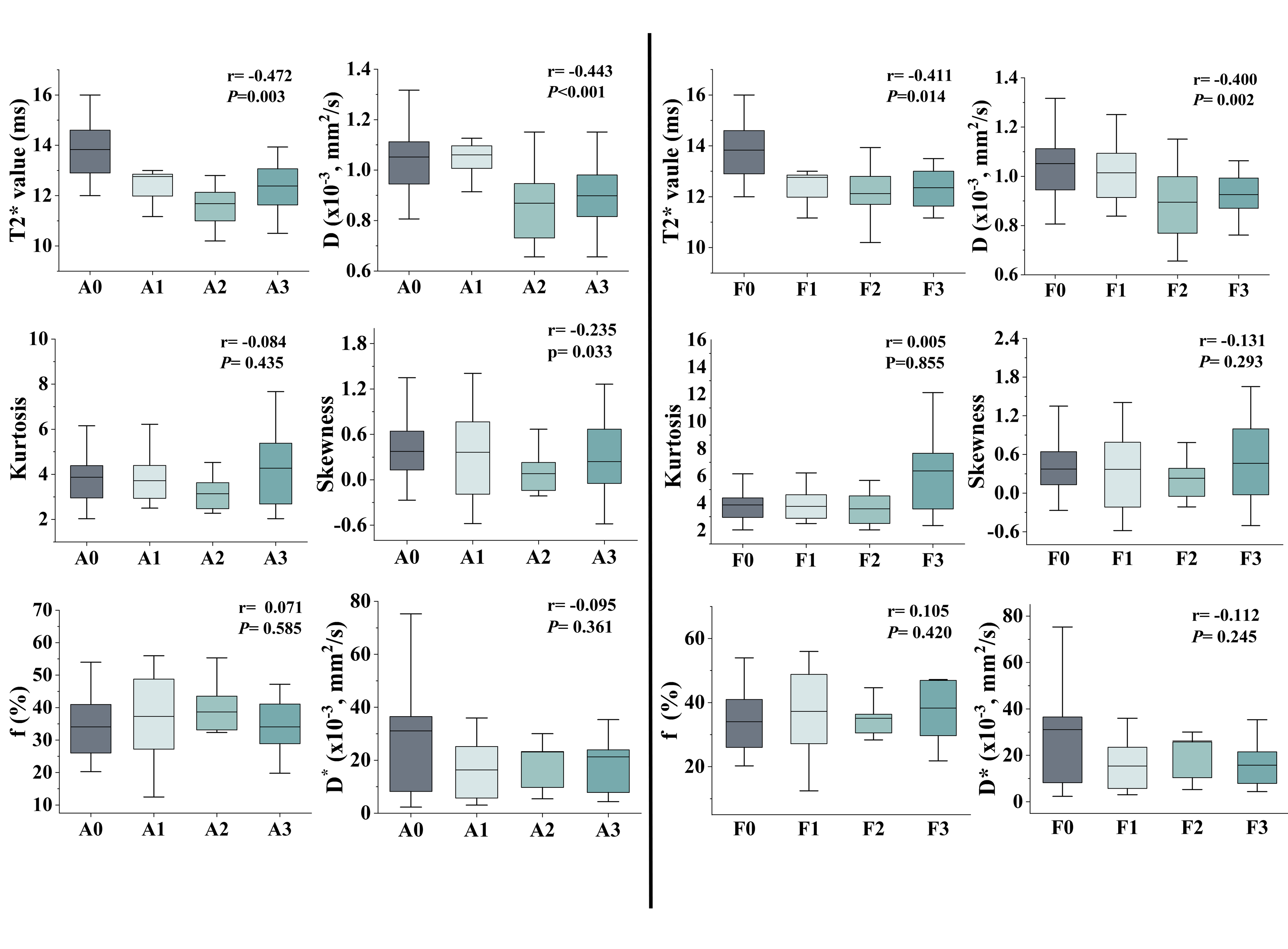

Supplement: Supplementary file 1 — Fig S1 Spearman correlation between other MRI parameters and fibrosis and inflammation. [file JMRI-55-1864-s001.tif]

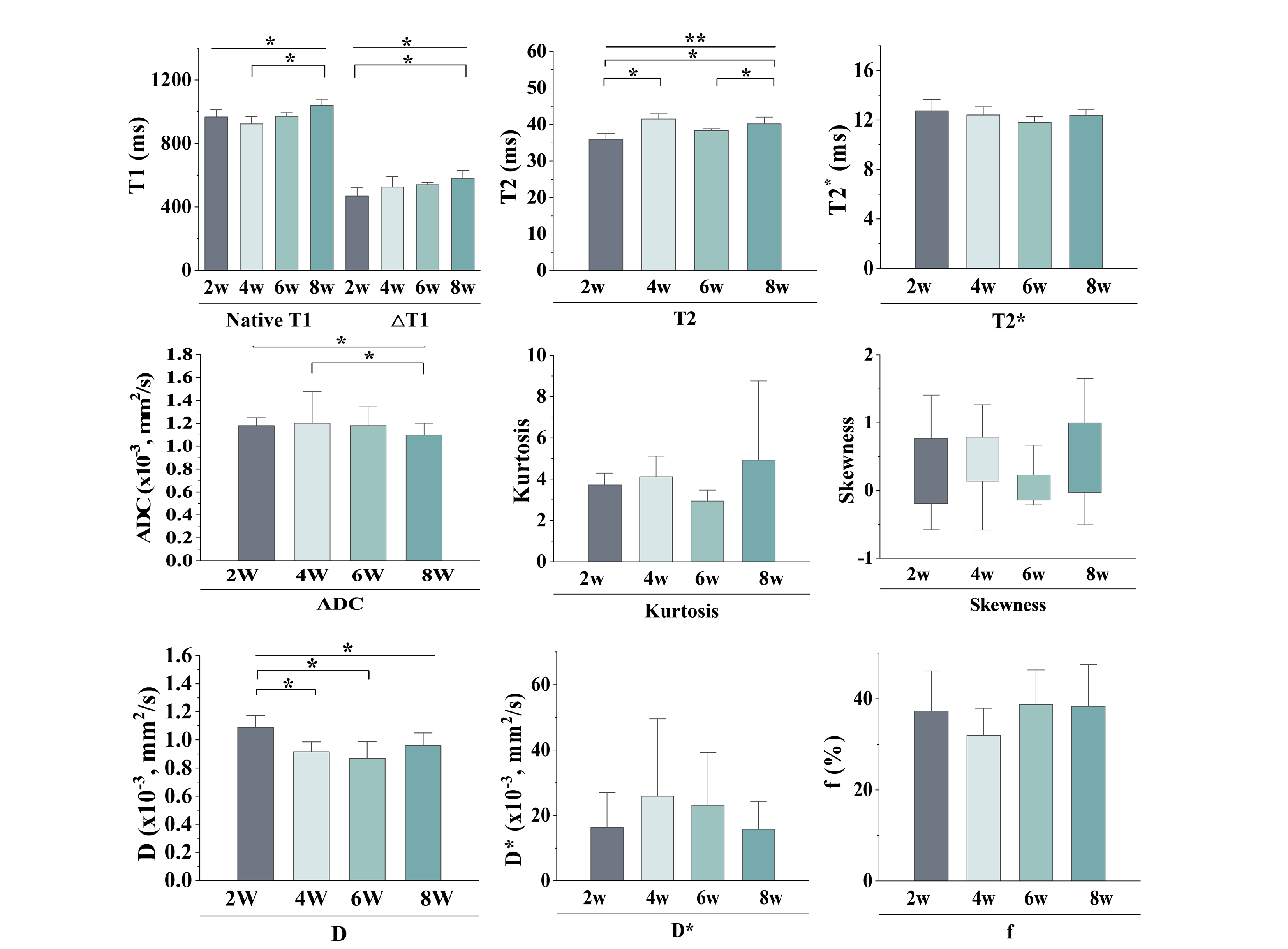

Supplement: Supplementary file 2 — Fig S2 Comparison of all MR parameters between the four different timepoints in the ligated liver group. *P < 0.05 and **P < 0.01. [file JMRI-55-1864-s002.tif]
